# Rederivation by Cryopreservation of a Paternal Line of Rabbits Suggests Exhaustion of Selection for Post-Weaning Daily Weight Gain after 37 Generations

**DOI:** 10.3390/ani10081436

**Published:** 2020-08-17

**Authors:** Jorge Daniel Juarez, Francisco Marco-Jiménez, Raquel Lavara, José Salvador Vicente

**Affiliations:** 1 Facultad de Zootecnia, Universidad Nacional Agraria de la Selva, Tingo María 10131, Peru; jorjua@posgrado.upv.es; 2 Instituto de Ciencia y Tecnología Animal, Universitat Politècnica de València, 46022 Valencia, Spain; fmarco@dca.upv.es (F.M.-J.); lavara.raquel@gmail.com (R.L.)

**Keywords:** selection programme, embryo vitrification, Gompertz growth curve, biobanking, reproductive performance

## Abstract

**Simple Summary:**

This study was conducted to evaluate the effect of a long-term selection for post-weaning daily weight gain after 37 generations, using vitrified embryos with 18 generational intervals to rederive two coetaneous populations, reducing or avoiding genetic drift, environmental and cryopreservation effects. This study reports that the selection programme had improved average daily weight gain without variations in adult body weight but, after 37 generations of selection, this trait seems exhausted.

**Abstract:**

Rabbit selection programmes have mainly been evaluated using unselected or divergently selected populations, or populations rederived from cryopreserved embryos after a reduced number of generations. Nevertheless, unselected and divergent populations do not avoid genetic drift, while rederived animals seem to influence phenotypic traits such as birth and adult weights or prolificacy. The study aimed to evaluate the effect of a long-term selection for post-weaning average daily weight gain (ADG) over 37 generations with two rederived populations. Specifically, two coetaneous populations were derived from vitrified embryos with 18 generational intervals (R19 and R37), reducing or avoiding genetic drift and environmental and cryopreservation effects. After two generations of both rederived populations (R21 vs. R39 generations), all evaluated traits showed some progress as a result of the selection, the response being 0.113 g/day by generation. This response does not seem to affect the estimated Gompertz growth curve parameters in terms of the day, the weight at the inflexion point or the adult weight. Moreover, a sexual dimorphism favouring females was observed in this paternal line. Results demonstrated that the selection programme had improved ADG without variations in adult body weight but, after 37 generations of selection, this trait seems exhausted. Given the reduction in the cumulative reproductive performance and as a consequence in the selection pressure, or possibly/perhaps due to an unexpected effect, rederivation could be the cause of this weak selection response observed from generation 18 onwards.

## 1. Introduction

Traits related to prolificacy for maternal lines, and feed conversion rate and carcass and meat quality for paternal lines, are commonly used in selection programmes in rabbits [1,2,3,4,5,6,7,8]. Some of them are difficult or expensive to measure (for instance, feed conversion rate trait), so correlated traits such as growth rate have been successfully used in selection [2,7,9,10,11,12,13,14]. Accurately, post-weaning daily weight gain has been estimated from 0.45 to 1.73 g/day (18 to 68 g) per generation in rabbit [2,8,11,12,15,16,17,18]. Body weight at slaughter time ranged in heritability from 0.12 to 0.67 as a consequence of environmental variability, the improvement of facilities, nutrition and feed systems, making it difficult to assess during the selection programme [7].

The success of rabbit selection programmes has been evaluated using a control population, in some cases from rederived cryopreserved embryos or by divergent selection [13,17,18,19,20,21]. Nevertheless, unselected control or divergent populations do not avoid genetic drift, while rederivation of animals by cryopreservation seems to affect phenotypic traits such as birth and adult weights or prolificacy, not only in animals born after transfer, but also their offspring [22,23]. Whether the phenotypic changes are only intragenerational, as a consequence of the embryonic stress response to cryopreservation and transfer, or transgenerational, as a consequence of heritable changes introduced at the epigenome level, is yet to be assessed [24,25]. However, cryopreservation offers the great advantage of being able to measure the genetic progress through generations, making evaluation possible in the same environment, facilities and feed diets of individuals separated by many generations [26]. 

A control population obtained by cryopreservation has rarely been used in selection experiments. In rabbits, it has been used to evaluate both the response to selection for litter size in maternal lines and for average daily weight gain in paternal lines [18,27]. García and Baselga [27] observed that the estimated responses obtained when using a rederived population of cryopreserved embryos or a mixed model that took into account the kinship matrix (genetic relationship) were different, suggesting that this latter model is less appropriate. Piles and Blasco [18] demonstrated that the selection for average daily gain between the 3rd–4th and 10th generations was successful and the response obtained was similar, using a rederived control population or a model with genetic relationships (0.62 to 0.65 g/day by generation). The latter study was performed with the paternal line used in this work. This study aimed to evaluate the effect of a long-term selection for average daily gain (37 generations) on commercial growth traits and Gompertz parameters, using two population rederived from vitrified embryos with 18 generational intervals to reduce or avoid genetic drift, environmental and cryopreservation effects.

## 2. Materials and Methods 

All the experimental procedures used in this study were performed following Directive 2010/63/EU EEC for animal experiments and reviewed and approved by the Ethical Committee for Experimentation with Animals of the Universitat Politeècnica de València, Spain (research code: 2015/VSC/PEA/00061).

### 2.1. Animals

**Figure 1:**
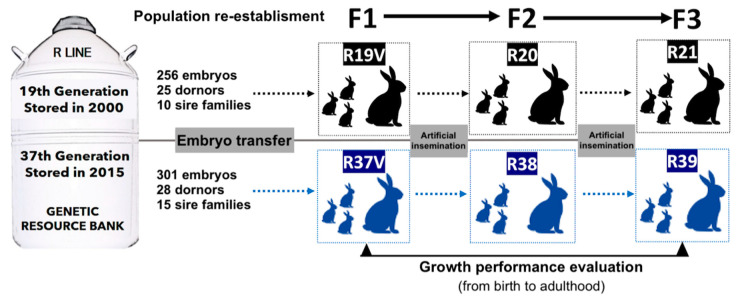
Experimental design: Two experimental progenies were developed from vitrified embryos stored in 2000 (19th generation of selection) and 2015 (37th generation of selection). All rabbits were identified and weighted at weaning and end of the fattening period to calculate the average daily gain. A sample of males and females were weighted weekly from birth to 20 weeks age in F1 y F3 generation to estimate Gompertz curve parameters.

A rabbit paternal line (R) selected at the Universitat Politècnica de València was used. This line was founded in 1989 from two closed paternal lines selected according to individual weight gain from weaning to end of fattening period (77 days old) during 12 and 9 generations [11]. Since then, the line has been selected for individual daily weight gain from 28 days (weaning) to 63 days of age (end of fattening phase). Animals from two different generations of selection were used. R19V population was rederived from 256 embryos of 25 donors belonging to ten different sire families of 18th generation vitrified in 2000. R37V population was rederived from 301 embryos of 28 donors belonging to 15 different sire families of 36th generation and they were vitrified in 2015. Both animal groups were transferred at the same time in 2015 (see details in Marco-Jiménez et al. [26]). Offspring were bred in non-overlapping generations during 2 generations (R20–R21 and R38–R39 from R19V and R37V, respectively). Figure 1 and Table 1 show the experimental design and the parents used to generate the offspring analysed in this study. 

The animals were housed at the Universitat Politècnica de València experimental farm in flat deck indoor cages (75 × 50 × 30 cm), with free access to water and commercial pelleted diets (minimum of 15 g of crude protein per kg of dry matter (DM), 15 g of crude fibre per kg of DM and 10.2 MJ of digestible energy (DE) per kg of DM). The photoperiod was 16 h of light and 8 h of dark, with a regulated room temperature between 14 °C and 28 °C.

### 2.2. Embryo Vitrification and Transfer to Population Rederivation

Five-hundred-and-fifty-seven embryos were used from donors belonging to R18 and R36 generations. Vitrification and transfer were described elsewhere [26,28,29]. Briefly, the vitrification was carried out in two steps at room temperature (approximately 20–22 °C). In the first step, embryos from each donor doe were placed for 2 min in an equilibrium solution consisting of 12.5% dimethyl sulfoxide (DMSO) and 12.5% of ethylene glycol (EG) in Dulbecco’s phosphate-buffered serum (DPBS) supplemented with 0.1% (*w*/*v*) of bovine serum albumin (BSA). In the second step, embryos were suspended for 1 min in the vitrification solution containing 20% DMSO and 20% EG in DPBS supplemented with 0.1% of BSA. Then, embryos suspended in vitrification medium were loaded into 0.125 mL plastic straws (ministraws, L’Aigle, France) and plunged directly into liquid nitrogen. After storage in liquid nitrogen, embryos were warmed and vitrification solution was removed, loading the embryos into a solution containing DPBS and 0.33 M sucrose for 5 min, followed by one bath in a solution of DPBS for another 5 min before transfer. Immediately after warming, the embryos were evaluated morphologically, and only embryos without damage in mucin coat or pellucid zone were transferred by laparoscopy into the oviduct of synchronised recipient females from a maternal line (Line A [29]) following the procedure described by Besenfelder and Brem [30] and García-Domínguez et al. [29]. The re-establishing of both populations to generate the 19th (R19V) and 37th (R37V) generation was described by Marco-Jiménez et al. [26].

### 2.3. Reproduction Management in Rederived and Filial Generations

Rederived rabbits were conducted in non-overlapping generations and the generation interval was approximately 9–10 months. The first reproductive cycle took place at ~5 months of age, and after kindling the new insemination was tried 10–12 days later. Insemination between relatives sharing a grandparent was avoided. Briefly, two ejaculates per male were collected in each replica using an artificial vagina. Ejaculates were diluted 1:5 with Tris-Citrate-Glucose extender and both motility and abnormal spermatozoa were assessed under phase optic at 200×. Only ejaculates with total motility higher than 70% and less than 30% of abnormal sperm were used. After semen evaluation, optimal ejaculates from each male were pooled and extended to 40 million/mL. All females were synchronised with 15UI eCG injected intramuscularly 48h before being inseminated with 0.5 mL of extended semen using a plastic curved pipette. Females were induced to ovulate by intramuscular injection of 1 µg of buserelin acetate at insemination time. Pregnancy was checked at 14 days from insemination and non-pregnant does were inseminated again at 21 days after the previous insemination. In addition, it was noted whether rabbits underwent a lactation–gestation overlap, classifying the reproductive status of their dams in four levels: offspring from primiparous does without overlapping (PD), offspring from primiparous lactating does (females that were pregnant while suckling their first litter, PLD), multiparous lactating does (females with more than one birth that were pregnant while suckling their litter, MLD) and multiparous non-lactating does (females from more than one parturition that were pregnant after lactation, MD).

### 2.4. Growing Traits Analysis

The offspring born of vitrified embryos were named R19V and R37V, the first filial generation (R20 and R38) and the second filial generation (R21 and R39). Individual weaning weight (WW, 30 days old), individual weight at end of the fattening period (EFW, 63 days old) and average daily weight gain (weight gained from day 28 to 63 divided by 33, ADG) during the fattening period were noted for all generations. To determine differences in growth curve, 76 and 113 rabbits from the rederived population (18 and 21 females and 16 and 21 males from R19V and R37V, respectively) and second filial generation (29 and 39 females and 18 and 27 males from R21 and R39, respectively) were identified at birth with chip and weighed weekly from born to 20 weeks old.

### 2.5. Statistical Analysis

A first analysis for growth traits was performed, attending to the generation interval of rederived population,

Y_ijklm_ = μ + P_i_ + R_j_ + MY_k_ + PR_ij_ + CO_l_ + Cov X_m_ + e_ijklm_ (1)

where Y_ijkl_ was the trait to analyse, µ was the general mean, P_i_ was the fixed effect of selection generation of rederived population R19 (R19V, R20, R21) and R37 (R37V, R38, R39), R_j_ was the fixed effect of reproductive status of the doe used in the WW analysis (PD, PLD, MLD and MD), M_Yk_ was the fixed effect of month-year in which the fattening period ended (39 levels), PR_ij_ was the effect of interaction between rederived population and reproductive status of the mothers used for WW analysis, CO_l_ was the random effect of common litter, Cov X_m_ was the covariate of the number born alive (BA) used for the WW trait or the covariate of WW used for weight at the end of the fattening period (EFW) and ADG traits and e_ijklm_ was the error term of the model.

A second analysis of growth traits to evaluate each filial generation (F1-R19V vs. R37V, F2-R20 vs. R38- and F3-R21 vs. R39) was performed using the mixed linear model described above:

Y_ijklm_ = μ + P_i_ + R_j_ + MY_k_ + PR_ij_ + CO_l_ + Cov X_m_ + e_ijklm_ (2)

where Pi was the fixed effect of filial generation (R19V and R37V or R20 and R38 or R21 and R38). In the WW analysis of the first filial generation, the effect of reproduction status of the mothers (R_j_) and the interaction (PR_ij_) were not included, all hosted females were multiparous non-lactating (MD) and the fixed effect month-year had 4 levels. In the analysis of filial generation 2, the fixed effect month-year (MY_k_) had 19 levels. For filial generation 3, MY_k_ fixed effect had 25 levels.

Finally, Gompertz curve parameters were estimated for each rabbit from R19V and R37V, and R21 and R39 for each rabbit:

X_jm_ = a_m_ exp[−b_m_ exp(−k_m_t)] + e_jm_ (3)

where X_jm_ is the body weight (BW) of m animal at t age (days); am, bm and km are the Gompertz growth curve parameters of m animal; and e_jm_ is the residual. As to the meaning of parameters, a can be interpreted as the mature BW maintained independently of short-term fluctuations, b is a timescale parameter related to the initial BW and k is a parameter related to the rate of maturing. The inflexion time (t_I_) is the moment when growth rate is maximum and is determined by t_I_ = (log e^b^)/k and inflexion weight (w_I_) were fixed at 0.37 of adult weight (a) by Gompertz curves.

A mixed linear model was fitted for the analysis of Gompertz growth curve parameters as follows:

Y_ijkl_ = μ + P_i_ + S_j_ + PS_ij_ + CO_k_ + CovB_l_ + e_ijkl_ (4)

where Y_ijkl_ was the Gompertz parameter, P_i_ is the fixed-effect population (R19V and R37V or R21V and R39V), S_j_ the fixed effect of the sex, PS_ij_ was the effect of interaction between population and sex, CO_k_ is the random effect of common litter, CovB_l_ was the weight at birth as a covariate and e_ijkl_ was the error term of the model. 

## 3. Results

### 3.1. Descriptive Growing Traits

A total of 2025 (2991 liveborn) animals from the three filial generations that finished fattening were obtained from 437 parturitions (27, 134 and 276 parturitions in the first, second and third generations respectively, Table 1). 

Average body weight at the end of fattening was 2.240 kg (EFW, 63 days old) and ADG was 43.95 g/day (Table 2). 

Mortality by rederived generations (R19V-R20-R21 vs. R37V-R38-R39) was 17.5% and 20.5% in the lactation period and 15.2% and 16.6% in the fattening period, respectively. For each filial generation, the mortality at the end of the lactation and fattening period reached levels of 3.3% and 19.6% for F1, 21.3 and 10.2 for F2 and 19.3% and 18.4% for F3, respectively. Data not shown in tables.

### 3.2. Growing Traits by Generation Interval of Rederived Populations. Selection Effect

Selection had a significant effect on studied traits and the estimated effects on WW, EFW and ADG were 0.031 ± 0.014 kg, 0.058 ± 0.013 kg and 1.55 ± 0.392 g/day (R37-R19, *p* < 0.05, Table 3). Moreover, reproductive status of does affected the WW, observing that the young mothers without lactation-gestation overlap (ND) had the lowest WW (0.632 ± 0.016 vs. 0.734 ± 0.019, 0.746 ± 0.015 and 0.718 ± 0.016 kg to PD, PLD, MLD and MD, respectively, *p* < 0.05, data not shown in tables). No interaction between selection generation of rederived population and reproductive status was found. MY fixed and common litter random effects were significant for all analyses (data not shown in tables). As expected, the warm months of July, August and September from each year had adverse effects on the traits studied.

### 3.3. Evolution of Growth Traits by Filial Generation

Weaning weight (WW) was not different in the first two filial generations (F1 and F2), and it was different and favourable to R39 at third filial generation (0.053 ± 0.022 and 0.066 ± 0.019, respectively, *p* < 0.05). On the contrary, the estimated effects on weight at the end of fattening (EFW) and ADG were always significant and favourable in each comparison to the last generations (Table 4). 

Reproductive status of does in F2 and F3 affected the WW. It was observed that the young mothers without lactation–gestation overlap had the lowest WW. No interaction between selection generation of rederived population and reproductive status was found. MY fixed were significant for all analyses, while common litter effect was not significant for EFW and ADG in F1 (R37V-R19V).

### 3.4. Estimated Growth Curves: F1 Rederived Populations (R19V and R37V)

No differences in Gompertz parameters were found for R19V and R37V population and sex (Table 5). 

**Figure 2:**
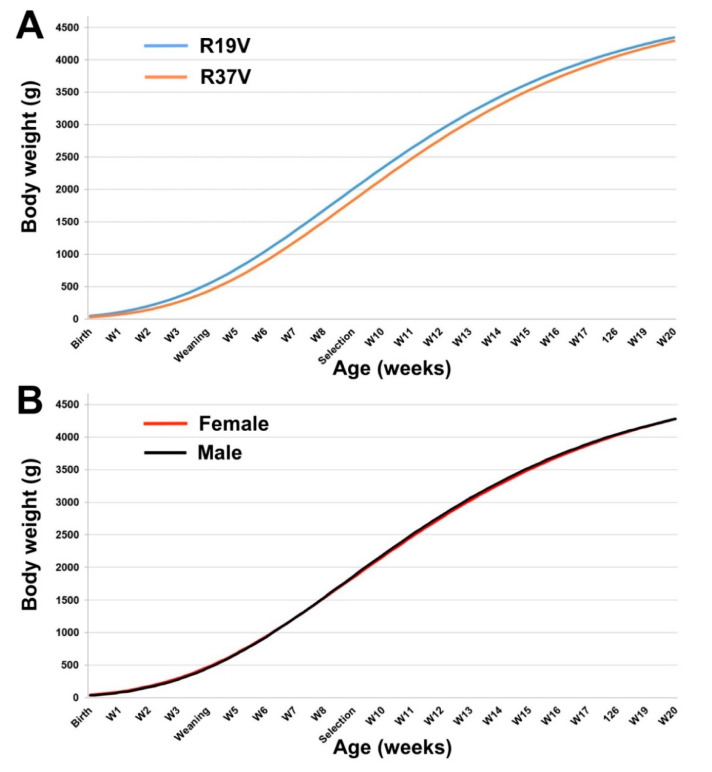
Growth curves from birth to 20-week-old between F1 rederived populations from R19V and R37V generations. V: Rederived from vitrified embryos. Growth curves were fitted using the Gompertz equation for (**A**) R19V and R37V generations and (**B**) sex.

Estimated growth patterns showed that both population and sex reached a maximum growth rate between 59 and 61 days old and with a weight about 1800 g. In addition, the estimated adult weights were between 4800 and 5000 g (Figure 2).

### 3.5. Estimated Growth Curves: F3 Rederived Population (R21 and R39)

**Figure 3:**
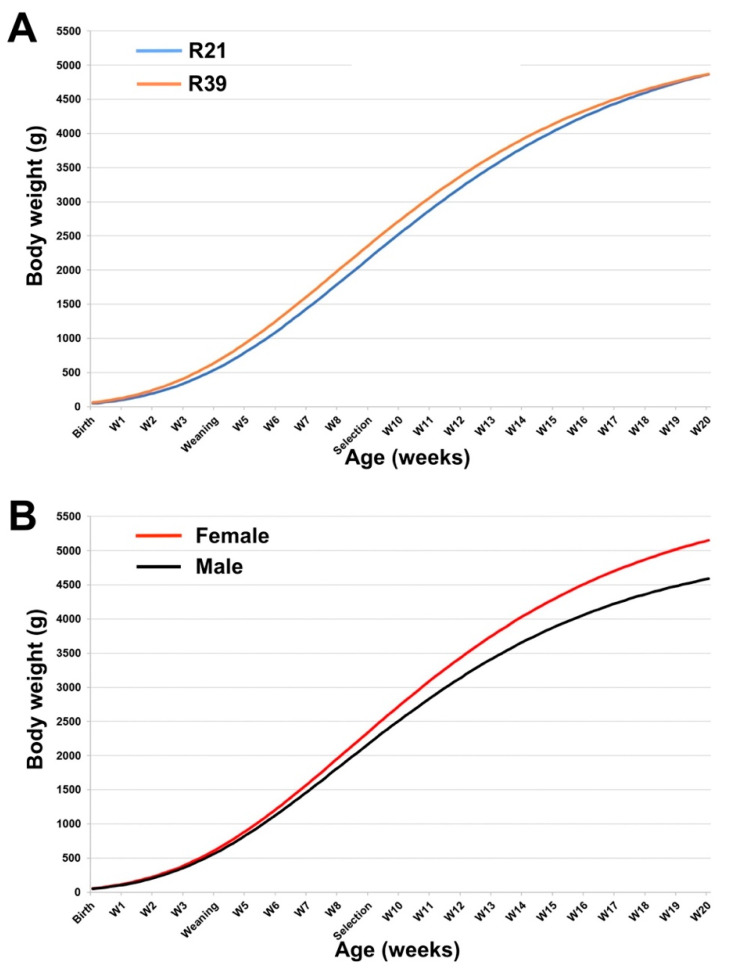
Growth curves from birth to 20-week-old between F3 filial generations from R21 and R39 generations. V: Rederived from vitrified embryos. Growth curves were fitted using the Gompertz equation for (**A**) R21 and R39 generations and (**B**) sex.

Gompertz parameters for R21 and R39 populations suggest no relevant differences between both populations (Table 6, Figure 3A). However, Gompertz parameters showed sexually dimorphic growth. Thus, males reached the inflexion point sooner (~3 days) and had a lower adult weight (Table 6, Figure 3B). Moreover, an interaction between population and sex was observed, suggesting a significant dimorphic pattern in R21 population as a consequence of a minor growth rate of R21 females (Figure S1).

## 4. Discussion

Rabbit meat programmes, like other animal breeding programmes, have based selection on the improvement of target criteria in controlled environments (e.g., litter size, BW daily gain, meat quality or feed efficiency [1,3,5,6,8,11,14,15,18,20,31,32,33,34]). Individual selection by ADG has been a common criterion for selecting rabbits in paternal lines, as it is effortless to record and has a moderate heritability (0.15–0.18), and favourable genetic correlation with the conversion index [12,14]. Related to this, response to selection for ADG has been estimated by comparison with a control population [2,8,17], or by divergent selection [12]. The response obtained by generation ranged between 0.56 g/day to 6 g/day [12,18]. Nevertheless, Magheni and Christensen [9] demonstrated an asymmetrical response to divergent selection, the response being greatest in the downward rather than in the upward lines, and therefore higher realised heritabilities were recorded in the downward than in upward lines. It is worth mentioning that in all studies, the response was lower than expected, attending to heritability estimates [7]. In our study, the average daily gain was compared between two rederived populations separated by 18 generations (approximately 15 years). This experimental approach reduces the influence of environmental effects, management, nutrition and even the rederivation procedure, as both populations were cryopreserved, transferred in the same host maternal line and reproductively conducted in the same way under the same environmental and feeding conditions. In this study, the response to the selection for ADG between the 19th and 37th generations was lowest compared to the results obtained in the same line between the 3–4th versus 11th generation [18]. These findings suggest that the response for ADG has been reduced across generations. Despite further research being required, we hypothesise that in part it would be as a consequence of an unfavourable effect on reproductive performance [35,36] and high mortality rates both during lactation (26% in Lavara et al. [37] and 17.5 and 20.5% in the present study to all R19 and R37 rederived generations, respectively) and the fattening period (15.2% and 16.6% to all R19 y R37 rederived generations, respectively), reducing the applied selection pressure and variability. On the other hand, rederivation from cryopreserved embryos could be a noneffective way to generate a control population [24,25]. All this evidence contributed to the response to selection being lower in the last 18 generations from a line with a total of 37 generations (0.09 g/day, Table 1). To the best of our knowledge, this is the first study carried out in rabbit after more than 37 generations of selection for ADG, if we take into account the lines from which it was founded in 1989. On the contrary, other studies have reported better results for genetic progression achieved after a few generations of selection [2,11,17,18,38].

In recent years, different studies have shown that assisted reproduction technologies and specifically embryo cryopreservation are not neutral [24,25]. Embryos are subjected to extreme environments in either recovery, cryopreservation and transfer that alter the gene expression, prenatal and postnatal development, and even modify reproductive performance (for example, focused on rabbit [22,23,24,25,39,40,41,42]). These effects not only modify the phenotype of those born from cryopreserved embryos, but their effects might also be affected and transferred to subsequent generations through heritable and non-heritable epigenetic changes [24,25]. First, in the first generation, newborns could be affected both by the vitrification transfer procedure and by maternal effects when developing in a different uterine and lactation environment, due to the mother in this experiment and the resulting litter size [26,43,44,45,46]. The second generation can be affected by the conditions in which parents developed. Finally, in the third generation, the heritable epigenetic effects generated by the rederived technique could emerge and become present. Lavara et al. [23] demonstrated that vitrification and transfer procedures involved in a rederivation programme for rabbit embryos have long-term consequences on rabbit growth patterns and might affect some growth-related traits in rabbits. Therefore, if the genetic artefacts introduced by the rederivation process were similar in both generations, evaluating their phenotypic differences from the third generation post-rederivation should be the most appropriate method with a minor cumulative genetic drift variance [47]. It has been observed that rederived populations showed differences in WW, end fattening weight and ADG as a consequence of selection. However, these differences were not constant after rederivation. Thus, the growth pattern was similar between the rabbits born from vitrified embryos (rederived populations, R19V and R37V) and the subsequent filial generations (R20 and R38). At weaning and end of fattening, the body weights were not different between generations. Only the first filial generation had a relevant difference for ADG (4.27 g/day), with a response of 0.237 g/day by generation. In the third filial generation (R21 and R39), all traits showed some progress as a result of the selection and the response was 0.113 g/day by generation. These deviations can be appreciated in the growth curve pattern, although they did not reach a significant difference either for the day or the weight at which the inflexion point is reached or the estimated adult weight. It should be noted that the inflexion point was slightly above the rabbit marketing age in Spain (56–58 days old) and near the selection age (63 days old). Blasco et al. [13], comparing a cryopreserved rederived population from 3rd and 4th generations from this paternal line with a 10th generation, concluded that estimated adult BW was increased by 7% after six generations, while other parameters of the Gompertz curve were scarcely affected by selection. In this context, the reduced sample size of rederived population advises caution regarding the conclusions of this study. 

The parameters and the resulting growth curve revealed a significant female sexual dimorphism pattern at the inflection point and estimated adult BW. Favourable female dimorphism was already being reported by several authors [48,49,50] in different breeds or synthetic lines of this species. Rall [48] described that females were larger than males by a proportion of 1.3:1, while Fuente and Rosell [50] reduce this ratio to 1.03. In this line, Blasco and Gómez [38] found no effect of sex in Gompertz parameters after 12 generations of ADG selection. Our data showed that sexual dimorphism was affected by ADG, going from a ratio of 1.0:1 to 1.14:1 in 18 generations. 

An undesirable consequence of selection for ADG could be the increment in adult BW, as it augments the costs of maintaining a parent population. Blasco et al. [13] found that Gompertz estimated adult BW increased with selection, whereas the parameters related to the slope of the curve practically did not change. In this circumstance, these latter authors predicted that male lines will become giant lines and the reproduction management will be more difficult, unless artificial insemination is used. An interesting finding of the study on long-term selection in these populations separated by 18 generations was that the selection for ADG did not change the estimated adult weight, although it seems to have reduced the adult female body weight (221 g, approximately −4% between R21 and R39 females). It is necessary to emphasise that from the parameters obtained in the populations directly rederived by vitrified embryo transfer (R19V and R37V), it was not possible to observe differences. This is probably due to the associated procedure and maternal effects derived from the transfer (smaller litter size and mother of the maternal line). Recent studies that demonstrated the incorporation of inheritable epigenetic marks [25] and the lack of knowledge as to whether or not they selectively affect certain genetic loci, could question the model used. Therefore, although rederived populations from cryopreserved embryos have some advantages, by optimising experimental facilities and reducing genetic drift this rederivation procedure could also contribute to distorting the control population [24,25]. Recently, García-Domínguez et al. [51] compared naturally conceived animals with progeny generated after embryo cryopreservation, observing transgenerational effects in differentially expressed transcripts and metabolites in hepatic tissue that could be associated with a lower adult weight of the rederived population. Another effect to take into account would be the differential storage time, although these studies were performed to analyse the effect on post-thaw embryo survival and pregnancy outcomes, but not on the liveborn development [52,53,54,55,56,57]. No studies have been performed to evaluate if the pattern of omics and phenotypic alteration was influenced by storage time.

As expected, the effect of the rabbit’s reproductive status (number of parturitions and overlap or not with lactation) on the WW was significant and unfavourable in the rabbits at first parity. Although the effect is restricted in young mothers without lactation and gestation overlap, our results were similar to those reported in earlier generations of R line [58], and in line with other paternal and maternal lines between primiparous and multiparous does [59,60,61]. This effect was related to the capacity of the doe to produce milk, which depends on the maturity of the female and the number of suckling kits [62,63,64,65]. In this regard, we observed that young females that became pregnant again after 10–12 days post-partum achieved a litter size weight at weaning similar to that of multiparous rabbits. Likewise, they showed a better health and physiological status than young mothers that were not pregnant after being inseminated. 

## 5. Conclusions

In conclusion, the present study describes the selection programme to improve post-weaning daily weight gain without increasing adult body weight in R line, but after 37 generation of selection this trait seems exhausted. A low accumulative reproductive performance or an unexpected rederived effect on growth traits might be the cause of this little selection response. A refoundation of line and selection for other criteria such as feed efficiency and maternal traits will be necessary.

## Figures and Tables

**Table 1:** The total number of parents used to generate the offspring analysed.

Rederived Population	Filial Generation	Generation	Females	Males	Total
19th	F1	R19V	14	11	**25**
	F2	R20	22	7	**29**
	F3	R21	50	16	**66**
		**Total**	**86**	**34**	**120**
37th	F1	R37V	11	10	**21**
	F2	R38	26	13	**39**
	F3	R39	78	18	**96**
		**Total**	**122**	**41**	**163**

V: Rederived from vitrified embryos.

**Table 2:** Number of young rabbits weighted from weaning to end fattening period and average and standard deviation to birth alive (BA), weaning weight (WW), weight at end fattening (EFW) and average daily weight gain (ADG).

Trait	Generation						
	R19V	R37V	R20	R38	R21	R39	Total
BA	3.4 ± 1.40	5.8 ± 3.30	6.7 ± 2.75	6.6 ± 2.95	6.8 ± 2.41	7.4 ± 3.16	6.8 ± 2.94
WW(Kg)	0.75 ± 0.236	0.64 ± 0.139	0.72 ± 0.162	0.72 ± 0.171	0.69 ± 0.165	0.71 ± 0.173	0.71 ± 0.171
EFW(Kg)	2.20 ± 0.302	2.17 ± 0.249	2.24 ± 0.279	2.33 ± 0.309	2.17 ± 0.323	2.25 ± 0.314	2.24 ± 0.313
ADG(g/day)	43.8 ± 5.72	46.3 ± 5.57	44.2 ± 5.98	46.9 ± 6.81	42.0 ± 7.08	43.6 ± 6.37	44.0 ± 6.72
Animals	42	52	285	347	489	810	2025

V: Rederived from vitrified embryos.

**Table 3:** Effect of selection for growth rate on weaning weight (WW, kg), weight at end of fattening period (EFW, kg) and average daily weight gain (ADG, g/day) from two rederived population after 18 generations.

Rederived Population	n	WW (kg)	EFW (kg)	ADG (g/day)
19th (R19V+R20+R21)	816	0.692 ± 0.014 ^b^	2.219 ± 0.013 ^b^	43.85 ± 0.375 ^b^
37th (R37V+R38+R38)	1209	0.723 ± 0.012 ^a^	2.277 ± 0.011 ^a^	45.41 ± 0.324 ^a^
Covariate effect		−0.028 ± 0.0024 ***	1.35 ± 0.029 ***	10.58 ± 0.847 ***

n: number of young rabbits. V: Rederived from vitrified embryos. Data are expressed as least squared mean ± standard error of means. ^a,b^ Values with different superscripts in column differ significantly (*p* < 0.05). Significance of estimated value of covariates (*** *p* < 0.001).

**Table 4:** Differences of least square means of weaning weight (WW, Kg), weight at end of fattening period (EFW, kg) and average daily weight gain (ADG, g/day) from filial generations. V: Rederived from vitrified embryos.

Trait	Generation		
	F1 (R37V-R19V)	F2 (R38-R20)	F3 (R39-R21)
WW (Kg)	0.043 ± 0.066	−0.040 ± 0.025	0.066 ± 0.019 **
EFW (Kg)	0.135 ± 0.056 *	0.062 ± 0.020 **	0.056 ± 0.018 **
ADG (g/day)	4.270 ± 1.578 *	1.658 ± 0.606 **	1.468 ± 0.544 **
Covariate effect			
BA(WW)	−0.028 ± 0.0122 *	−0.030 ± 0.0038 ***	−0.027 ± 0.0030 ***
WW(EFW)	1.20 ± 0.120 ***	1.33 ± 0.046 ***	1.36 ± 0.039 ***
WW(DG)	5.60 ± 3.581	10.4 ± 1.35 ***	11.0 ± 1.13 ***

Significance of estimated effects and covariates (* *p* < 0.05, ** *p* < 0.01, *** *p* < 0.001).

**Table 5:** Gompertz curve parameters from R19V and R37V generations.

Group	Gompertz Parameters				
	a	b	k	t (days)	W (g)
**Rederived Population**					
R19V	4906 ± 133	4.62 ± 0.269	0.026 ± 0.001	60.6 ± 2.33	1815 ± 49
R37V	4905 ± 128	5.08 ± 0.288	0.026 ± 0.001	62.5 ± 2.46	1815 ± 47
**Sex**					
Female (F)	4939 ± 113	4.75 ± 0.211	0.025 ± 0.001	62.1 ± 1.84	1828 ± 42
Male (M)	4872 ± 114	4.93 ± 0.210	0.026 ± 0.001	61.0 ± 1.84	1803 ± 42
**Rederived Population × Sex**					
R19V × F	4887 ± 170	4.64 ± 0.297	0.030 ± 0.002	60.6 ± 2.63	1808 ± 63
R19V × M	4925 ± 168	4.57 ± 0.292	0.030 ± 0.002	60.6 ± 2.58	1822 ± 62
R37V × F	4992 ± 159	4.85 ± 0.306	0.030 ± 0.002	63.6 ± 2.66	1847 ± 59
R37V × M	4818 ± 155	5.29 ± 0.303	0.030 ± 0.002	61.4 ± 2.63	1783 ± 57

V: Rederived from vitrified embryos. ^a,b^: Different superscript between columns indicate statistical differences (*p* < 0.05). a: mature body weight; b: a timescale parameter related to the initial body weight; k: growth velocity; t: Inflexion age; w: weight at inflexion. LSM ± SEM: least square mean ± standard error of means.

**Table 6:** Gompertz curve parameters from R21 and R39 generations.

Group	Gompertz Parameters				
	a	b	k	t (days)	W (g)
**Population**					
R21	5518 ± 154	4.77 ± 0.095 ^a^	0.026 ± 0.001	60.1 ± 1.22	2042 ± 56.9
R39	5400 ± 135	4.49 ± 0.083 ^b^	0.027 ± 0.001	56.9 ± 1.07	1998 ± 50.1
**Sex**					
Female (F)	5815 ± 113 ^a^	4.63 ± 0.070	0.026 ± 0.000 ^a^	60.1 ± 0.89 ^a^	2151 ± 41.7 ^a^
Male (M)	5102 ± 132 ^b^	4.64 ± 0.082	0.027 ± 0.001 ^b^	56.9 ± 1.05 ^b^	1888 ± 48.8 ^b^
**Rederived Population × Sex**					
R21 × F	5925 ± 172	4.68 ± 0.107	0.025 ± 0.001 ^a^	62.7 ± 1.37	2192 ± 63.6
R21 × M	5110 ± 195	4.86 ± 0.122	0.028 ± 0.001 ^b^	57.4 ± 1.56	1891 ± 72.1
R39 × F	5704 ± 153	4.57 ± 0.095	0.027 ± 0.001 ^b^	57.5 ± 1.22	2111 ± 56.5
R39 × M	5095 ± 171	4.42 ± 0.106	0.027 ± 0.001 ^b^	56.4 ± 1.37	1885 ± 63.3

^a,b^ Different superscript between columns indicate statistical differences (*p* < 0.05). a: mature body weight; b: a timescale parameter related to the initial body weight; k: growth velocity; t: inflexion age; w: weight at inflexion. LSM ± SEM: least square mean ± standard error of means.

## Supplementary Materials

The following are available online at https://www.mdpi.com/2076-2615/10/8/1436/s1, Figure S1: Gompertz growth curve from birth to 20-week-old for interaction between populations and sex of animals.

## Abbreviations

ADG	Daily weight gain
R	Rabbit paternal line
DM	Dry matter
DE	Digestible energy
DMSO	Dimethyl sulfoxide
EG	Ethylene glycol
DPBS	Dulbecco´s phosphate buffered serum
PD	Primiparous does
PLD	Primiparous lactating does
MLD	Multiparous lactating does
MD	Multiparous non-lactating does
WW	Weaning weight
EFW	End of the fattening period
BW	Body weight
R19V	Rederived animals from generation 19
R37V	Rederived animals from generation 37
R20	First filial generation from R19V generation
R21	Second filial generation from R19V generation
R38	First filial generation from R37V generation
R39	Second filial generation from R37V generation
