# Supplementary material for: Rederivation by Cryopreservation of a Paternal Line of Rabbits Suggests Exhaustion of Selection for Post-Weaning Daily Weight Gain after 37 Generations
Source: Animals (Basel). 2020 Aug 17;10(8):1436. doi: 10.3390/ani10081436 (PMC7460551; doi:10.3390/ani10081436)
Supplement: Supplementary file 1 [file animals-10-01436-s001.pdf]

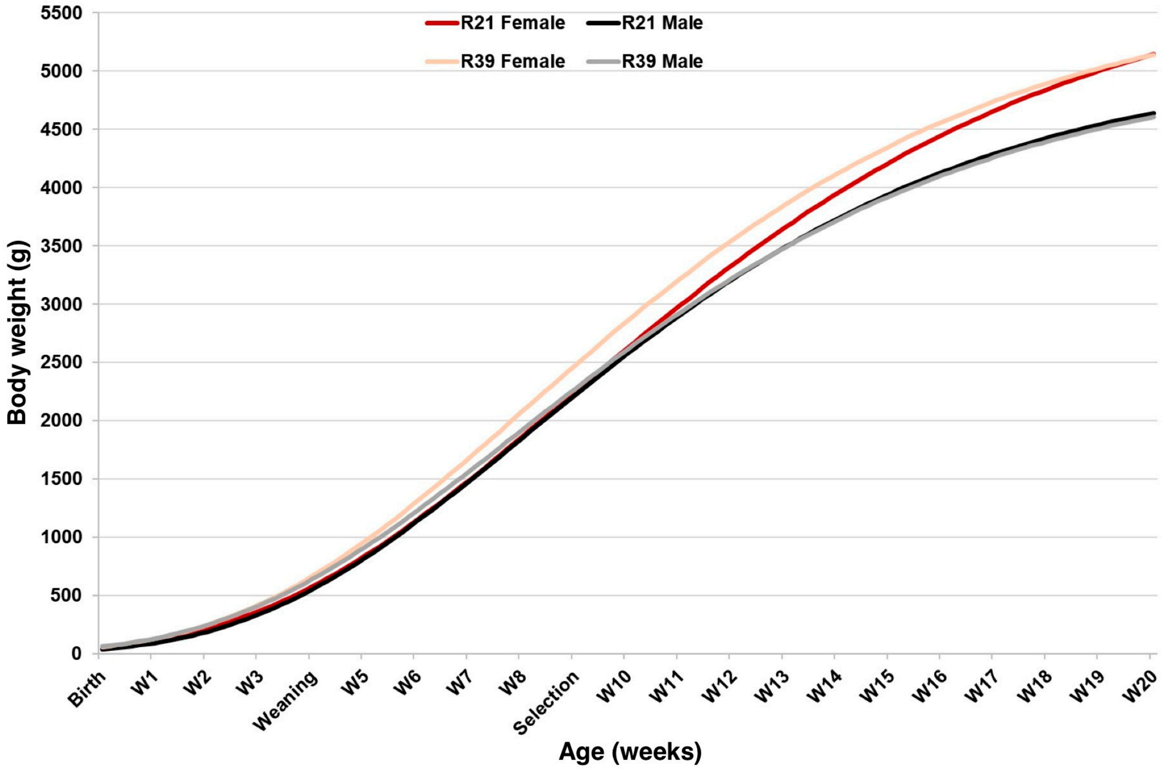

Figure S1. Gompertz growth curve from birth to 20-week-old for interaction between populations and sex of animals.
